# Fully Automated Detection of the Appendix Using U-Net Deep Learning Architecture in CT Scans

**DOI:** 10.3390/jcm13195893

**Published:** 2024-10-02

**Authors:** Betül Tiryaki Baştuğ, Gürkan Güneri, Mehmet Süleyman Yıldırım, Kadir Çorbacı, Emre Dandıl

**Affiliations:** 1 Department of Radiology, Medical Faculty, Bilecik Şeyh Edebali University, Bilecik 11230, Türkiye; 2 Department of General Surgery, Medical Faculty, Bilecik Şeyh Edebali University, Bilecik 11230, Türkiye; gurkanguneri@gmail.com; 3 Department of Sogut Vocational School, Computer Technology, Bilecik Şeyh Edebali University, Bilecik 11600, Türkiye; mehmets.yildirim@bilecik.edu.tr; 4 Department of General Surgery, Bilecik Osmaneli Mustafa Selahattin Çetintaş Hospital, Bilecik 11500, Türkiye; dr.kadircorbaci@gmail.com; 5 Department of Computer Engineering, Faculty of Engineering, Bilecik Seyh Edebali University, Bilecik 11230, Türkiye

**Keywords:** appendix detection, deep learning, medical imaging, segmentation, U-Net architecture

## Abstract

**Background:** The accurate segmentation of the appendix with well-defined boundaries is critical for diagnosing conditions such as acute appendicitis. The manual identification of the appendix is time-consuming and highly dependent on the expertise of the radiologist. **Method:** In this study, we propose a fully automated approach to the detection of the appendix using deep learning architecture based on the U-Net with specific training parameters in CT scans. The proposed U-Net architecture is trained on an annotated original dataset of abdominal CT scans to segment the appendix efficiently and with high performance. In addition, to extend the training set, data augmentation techniques are applied for the created dataset. **Results:** In experimental studies, the proposed U-Net model is implemented using hyperparameter optimization and the performance of the model is evaluated using key metrics to measure diagnostic reliability. The trained U-Net model achieved the segmentation performance for the detection of the appendix in CT slices with a Dice Similarity Coefficient (DSC), Volumetric Overlap Error (VOE), Average Symmetric Surface Distance (ASSD), Hausdorff Distance 95 (HD95), Precision (PRE) and Recall (REC) of 85.94%, 23.29%, 1.24 mm, 5.43 mm, 86.83% and 86.62%, respectively. Moreover, our model outperforms other methods by leveraging the U-Net’s ability to capture spatial context through encoder–decoder structures and skip connections, providing a correct segmentation output. **Conclusions:** The proposed U-Net model showed reliable performance in segmenting the appendix region, with some limitations in cases where the appendix was close to other structures. These improvements highlight the potential of deep learning to significantly improve clinical outcomes in appendix detection.

## 1. Introduction

The integration of artificial intelligence (AI) into medical diagnostics has transformed disease detection and treatment planning, improving both accuracy and efficiency. AI technologies, particularly deep learning algorithms, such as convolutional neural networks (CNN), have demonstrated remarkable potential in interpreting complex medical images and analyzing extensive clinical data. These advances have been crucial in several medical fields, including oncology, cardiology, and neurology, where AI systems have matched or exceeded human diagnostic performance [1,2].

Currently, AI-based approaches proposed for medical imaging can be divided into two subgroups: machine learning-based methods and advanced neural network-based methods such as deep learning [3]. AI, particularly deep learning models, has been instrumental in transforming diagnostics in many medical fields [4]. On the other hand, the development and application of Explainable AI (XAI) in medical diagnostics has gained considerable momentum in recent years. By providing interpretable results, XAI systems have been instrumental in building the trust and acceptance of AI tools among healthcare professionals. In addition, AI-based and XAI-based decision support systems play a critical role in these systems by providing clinicians with transparent and interpretable decision paths [5,6,7,8]. With the increasing integration of XAI, the adoption of AI systems in clinical practice would continue to grow, driven by both improved accuracy and interpretability of AI predictions [9]. Incorporating these advanced AI tools, such as deep learning-based architectures for appendix detection, into diagnostic workflows holds great promise for improving the speed, accuracy, and reliability of medical diagnoses.

Appendicitis, a common and potentially life-threatening condition, is one area where AI-driven diagnostic tools can make a significant impact. The timely and accurate diagnosis of appendicitis is crucial, as delays can lead to severe complications such as perforation and sepsis. While clinical examination and imaging techniques such as computed tomography (CT) scans are traditionally used for diagnosis, these methods are often subjective and highly dependent on clinician expertise [10].

Deep learning has increasingly become a critical tool in the medical field, offering advanced solutions for diagnosis, treatment planning, and patient care [4]. In recent years, several studies have shown that AI models, including machine learning and deep learning techniques, can improve diagnostic accuracy and support clinical decision-making [11,12,13]. AI, particularly deep learning, has shown promise in appendicitis diagnosis, with several studies highlighting the advantages of CNN-based models in analyzing medical images. Recent studies have underscored the effectiveness of AI in enhancing appendix diagnosis [14,15]. A systematic review by Issaiy et al. [16] highlighted the use of different AI algorithms, including deep learning models such as CNN, in diagnosing acute appendicitis. These models often outperform traditional diagnostic methods in identifying appendix from medical images like CT scans. In a recent study, Liang et al. [17] developed a deep learning model combined with radiomics to differentiate between complicated and uncomplicated acute appendicitis using pelvic CT scans. Their model outperformed radiologists, suggesting improved diagnostic performance. In another study, Marcinkevics et al. [18] focused on pediatric appendicitis and used machine learning models such as logistic regression, random forests, and generalized boosted regression models to predict the diagnosis and severity of appendicitis. These models were trained and validated using clinical data, demonstrating that AI can effectively aid in the diagnosis and management of pediatric appendicitis. Rajpurkar et al. conducted a study using a 3D deep learning model to diagnose appendicitis. They used an approach that exploits volumetric data from CT scans to improve diagnostic accuracy. The model demonstrated high sensitivity and specificity, outperforming traditional diagnostic methods and representing a significant advance in automated appendicitis detection [19]. These studies and applications highlight the significant advances and contributions of AI in the detection and diagnosis of appendicitis. However, there remains a gap in the previous works regarding fully automated segmentation of the appendix, particularly using deep learning architectures like U-Net.

Deep learning is extensively used in medical imaging and diagnostics, where CNNs analyze medical images [20]. For instance, deep learning models such as U-Net are used for image segmentation tasks in radiology, significantly enhancing diagnostic accuracy [19]. The U-Net deep learning architecture has many applications, including the detection of brain tumors [21], segmentation of retinal layers in optical coherence tomography images [22], breast cancer detection [23], and liver segmentation [24]. These examples highlight the versatility and effectiveness of the U-Net model in various medical imaging tasks, underscoring its importance in enhancing diagnostic accuracy and improving patient care. Despite these promising results, several challenges persist in optimizing deep learning models and AI methods for appendix detection. Factors such as data quality, model complexity, and the integration of multi-modal data significantly impact the performance of deep learning methods. Addressing these challenges through strategies like data augmentation, advanced model architectures, and rigorous model optimization is essential for maximizing the potential of deep learning in medical diagnostics [25,26].

In this study, we propose a U-Net-based architecture specifically designed for appendix segmentation in CT scans. The U-Net model is widely known for its encoder–decoder structure, which allows the precise localization and segmentation of complex structures in images. In the U-Net architecture, skip connections between the encoder and decoder layers help preserve spatial information and improve segmentation accuracy [27], which is essential for distinguishing the appendix from surrounding tissue. Our U-Net model has been designed with specific training parameters and data augmentation techniques to handle variability in CT scan quality and anatomy. These features are designed to optimize the model’s performance for the challenging task of appendix segmentation, which is often complicated by the small size and unclear boundaries of the appendix in medical images. To the best of our knowledge, there are almost no studies on appendix segmentation from CT scans. This study proposes U-Net deep learning architecture using the original dataset in appendix segmentation and discusses potential improvements to enhance diagnostic accuracy and clinical outcomes. This study provides several important contributions to the field of automated appendix detection and segmentation:

1. We have developed a U-Net model specifically tailored for appendix segmentation in CT scans, addressing a significant gap in the state-of-the-art scans. The architecture builds on the strengths of U-Net, using special training parameters and data enhancement techniques to cope with the variations in image quality and anatomy complexity;
2. The proposed model is trained on an original annotated dataset of abdominal CT scans and evaluated using key metrics such as DSC, VOE, ASSD, and HD95. These metrics demonstrate the reliable performance of the model in accurately segmenting the appendix, with a particular focus on minimizing false positives and false negatives;
3. This study employs hyperparameter optimization techniques to fine-tune the U-net architecture to ensure the highest possible segmentation performance. In addition, data augmentation strategies are applied to expand the training set and improve the model’s ability to generalize across different CT scan conditions;
4. While the model demonstrates high segmentation performance, we discuss potential limitations, particularly in cases where the appendix is close to other anatomical structures. We suggest directions for future improvement to enhance the diagnostic accuracy and clinical utility of the system.

The subsequent sections of the paper are organized as follows. The details of the original dataset generated for this study, the data augmentation procedures, and the methodology of the proposed system are described in Section 2. Section 3 deals with the results of the experimental analyses. The evaluation of the obtained results, the comparison with previous studies and the comparison with state-of-the-art methodology are discussed in Section 4. The study is concluded with inferences and focused future work in the last section.

## 2. Materials and Methods

**Figure 1:**
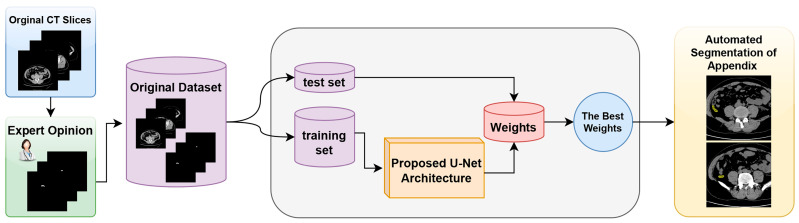
Block diagram of the proposed detection system for the fully automated detection of appendix region in CT scans.

The proposed model and the general structure of the study are shown in the block diagram in Figure 1. The methodology of the proposed approach consists of three main phases. The first phase is data collection and dataset preparation. In this phase, the necessary data are collected and a dataset for the proposed U-Net model is created. The second phase involves the structuring of the proposed U-Net deep learning model and the preparation of the working environment. In this phase, the architecture of the model is determined and the necessary software and hardware infrastructure is set up. The third phase is the automatic segmentation of the appendix region using the best weights obtained from the training processes. In this phase, the model is tested, the weights that give the best performance are determined, and the success of the model in the segmentation of the appendix region with these weights is evaluated.

A. Dataset

In this study, an original dataset was prepared for appendix detection and segmentation from CT scans using the proposed U-Net-based deep learning architecture. CT examinations were performed using a 64-MDCT scanner (multi-slice CT Aquillion 64; Toshiba). The imaging protocol included the following parameters: a slice thickness of 2.5 mm, a reconstruction interval of 0.777 mm, a gantry rotation time of 0.6 s, a tube voltage of 120 kV, and a tube current of 200 mA. The field of view was 40 to 50 cm. During CT imaging, slices were obtained from the upper abdomen to the lower abdomen with the patient in the supine position.

The original dataset consisted of CT scans of 299 patients who presented to the Bilecik Training and Research Hospital with abdominal pain and suspected acute appendicitis. These patients were assessed for demographic and laboratory characteristics and the presence or absence of appendicitis. As a result of the evaluations, 140 of these patients had a normal appendix, while the remaining 159 patients were diagnosed with acute appendicitis. Of the patients included in the dataset, 190 were male and the remaining 109 were female, with ages ranging from 18 to 91 years. The patients’ radiological images were obtained between July 2021 and April 2024. In addition, it was confirmed that there was no ethical problem in conducting this study with Decision Number 13 of the 8th meeting of Bilecik Seyh Edebali University Non-Interventional Clinical Research Ethics Committee on 5 December 2023. In addition, patients who have been treated at Bilecik Training and Research Hospital have agreed to provide their data for research purposes.

**Figure 2:**
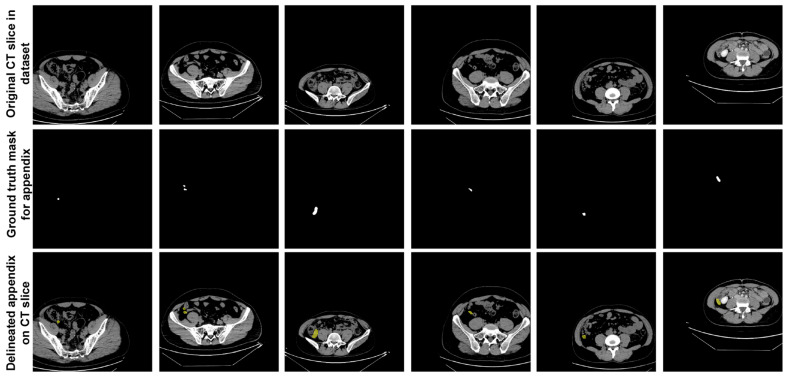
Sample slices from the dataset indicating the annotated appendix regions with GT masks.

In the dataset, expert physicians annotated the appendix regions with ground truth (GT) masks in these CT scans, using ITK-SNAP software [28], and the annotations were stored in NIfTI format. During the data preparation phase, axial slices identified by the expert physicians as containing the appendix were extracted. This process resulted in 672 slices from patients with appendicitis and 748 slices from healthy individuals, giving a total of 1420 slices extracted. These annotated slices formed the basis of the dataset used to train and evaluate the proposed U-Net deep learning model. Figure 2 shows sample slices from the dataset, indicating the annotated appendix regions with GT masks. This dataset, with its detailed annotation and diverse sample population, provided a robust approach for training the U-Net model to accurately segment the appendix in CT images.

B. Proposed Methodology

In this study, a deep learning model based on the U-Net architecture was proposed for detecting appendix areas on axial CT scans. The U-Net model is widely used in medical image processing, especially for biomedical image segmentation [29,30,31]. It has demonstrated its effectiveness in various medical applications by accurately classifying each pixel in an input image to delineate different tissues and structures. The U-Net architecture consists of two parts. The structure of the U-Net can be summarized for each key component in the contracting path (encoder) and the expansive path (decoder). The first part is the contracting path, which serves as the encoder [19]. In the encoder part, convolution, padding, and pooling operations are performed to compress the data into feature representations [32]. For each layer *Le* in the encoder, the output $O_{Le}^{en}$ is given Equation (1). The encoder extracts features and reduces spatial resolution while segmenting the image.

(1) $$O_{Le}^{en}=ReLU(Conv(O_{Le-1}^{en}))$$

where $O_{Le}^{en}$ represents the feature maps after layer *Le* in the encoder.

The second part is the expanding path, which serves as the decoder. In the decoder part, deconvolution, padding, and pooling operations are performed to expand the data, and the output from each corresponding level of the encoder is added at each layer to bring the data back to its original input size [33]. On the other hand, for each layer *Ld*, the output $O_{Ld}^{de}$ is shown in Equation (2). In the expansive path, the feature maps are upsampled using deconvolution (transposed convolution).

(2) $$O_{Ld}^{de}=ReLU(DeConv(O_{Ld-1}^{de}))$$

where $O_{Ld}^{de}$ represents the feature maps after layer *Ld* in the decoder.

Skip connections are a key feature of the U-Net architecture, allowing information to flow from the encoder to the decoder at corresponding levels [34]. This is a concatenation operation between the feature maps from the encoder and the decoder at each layer [27]. The skip connection is shown in Equation (3).

(3) $$O_{Ld}^{de}=concat(O_{Ld}^{de},\;O_{nL-Le}^{en})$$

where *nL* is the number of layers in the encoder.

In U-Net architecture, the final layer performs a convolution operation to obtain the segmented data. The final layer $O_{final}\left(x,y\right)$ applies a 1×1 convolution to map the feature maps to the desired number of output classes, as seen in Equation (4).

(4) $$O_{final}\left(x,y\right)=sigmoid({Conv}_{1\times 1}\;(O_{Ld}^{de}))$$

**Figure 3:**
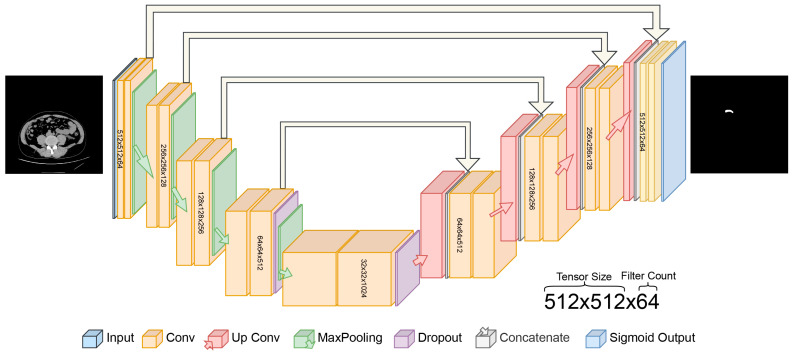
Proposed U-Net deep learning architecture for automated detection of appendix.

In this study, the proposed U-Net architecture for automatic appendix detection and segmentation from CT scans is shown in Figure 3. The proposed U-Net model for appendix segmentation differs significantly from the models commonly used in the past, mainly because it uses sigmoid output instead of softmax in the final layer. In addition, the contracting path was repeated four times in the proposed U-Net model. The specific architecture of the U-Net, designed for segmentation, features a symmetric encoder–decoder structure with skip connections, which is particularly effective at capturing the fine details needed to segment small anatomical structures such as the appendix [35]. In contrast, state-of-the-art models are not as well-adapted to this task and often require additional layers or modules to achieve a similar performance [36,37].

C. Key Performance Metrics

In this study, the performance of the proposed deep learning model for automatic appendix segmentation is evaluated using a number of important key metrics. The performance of the proposed model was measured using overlap-based, distance-based and pixel-based key metrics. Furthermore, these metrics were used to measure the similarity between the segmented areas ($A_{S})$ by the proposed U-Net-based deep learning model and the expert annotations ($A_{E}$) as a ground truth mask for the appendix. The Dice Similarity Coefficient (DSC), in Equation (5), is commonly used to compare the predicted segmentation result by the proposed method with the ground truth mask by the expert. On the other hand, the Volumetric Overlap Error (VOE), in Equation (6), is used to evaluate the dissimilarity between two volumes and measure how much the predicted segmentation deviates from the ground truth segmentation. The Average Symmetric Surface Distance (ASSD), a distance-based key metric, measures the average distance between the surfaces of the predicted segmentation and the ground truth and is given in Equation (7). The Hausdorff Distance (HD), given in Equations (8)–(10), is a measure of the distance between the predicted segmentation boundary and the ground truth segmentation boundary. Hausdorff Distance 95 (HD95) uses this by taking the 95th percentile of the distances between boundary points rather than the maximum distance. Precision (PRE), in Equation (11), refers to the ratio of correctly predicted positive pixels (true positive, TP) to the total number of pixels predicted as positive (true positive + false positive, TP + FP) by the model in the segmentation tasks. Recall (REC) represents the proportion of correctly predicted positive pixels (TP) out of all true positive pixels (TP + false negative, FN) in the ground truth and is shown in in Equation (12).

(5) $$\mathrm{DSC}\;(A_{E},A_{S})=\frac{2\left|A_{E}\cap A_{S}\right|}{\left|A_{E}\right|\cup \left|A_{S}\right|}\times 100$$

(6) $$\mathrm{VOE}\;\left(A_{E},A_{S}\right)=\left(1-\frac{\left|A_{S}\cap A_{E}\right|}{\left|A_{S}\right|+\left|A_{E}\right|-\left|A_{S}\cup A_{E}\right|}\right)\times 100$$

(7) $$\mathrm{ASSD}\;(A_{E},A_{S})=\frac{1}{\left|A_{E}\right|+\left|A_{S}\right|}\times \left(\sum_{x\in A_{S}}\underset{y\in A_{E}}{\min}d\left(x,y\right)+\sum_{y\in A_{S}}\underset{x\in A_{E}}{\min}d\left(y,x\right)\right)$$

(8) $$hd(A_{S},A_{E})=\underset{\mathrm{x\epsilon}A_{S}}{\max}\underset{\mathrm{y\epsilon}A_{E}}{\min}{\left\|x-y\right\|}_{2}$$

(9) $$hd(A_{E},A_{S})=\underset{\mathrm{x\epsilon}A_{E}}{\max}\underset{\mathrm{y\epsilon}A_{S}}{\min}{\left\|x-y\right\|}_{2}$$

(10) $$HD\left(A_{S},A_{E}\right)=max\left(hd(A_{S},A_{E}),hd(A_{E},A_{S}\right))$$

(11) $$\mathrm{PRE}=\frac{\mathrm{TP}}{\mathrm{TP}+\mathrm{FP}}\times 100$$

(12) $$\mathrm{REC}=\frac{\mathrm{TP}}{\mathrm{TP}+\mathrm{FN}}\times 100$$

## 3. Results

In this study, comprehensive experiments are conducted to evaluate the performance of the proposed U-Net-based deep learning model for appendix segmentation on CT scans from an original dataset. The experimental analyses reveal the performance and challenges of the proposed model in appendix segmentation. The experimental analyses were performed on a workstation equipped with an NVIDIA RTX 3060 GPU, 32 GB RAM, and an Intel i5-13400T CPU. The operating system used was Ubuntu 22 and the scripts were developed using Python with TensorFlow and Keras frameworks.

**Figure 4:**
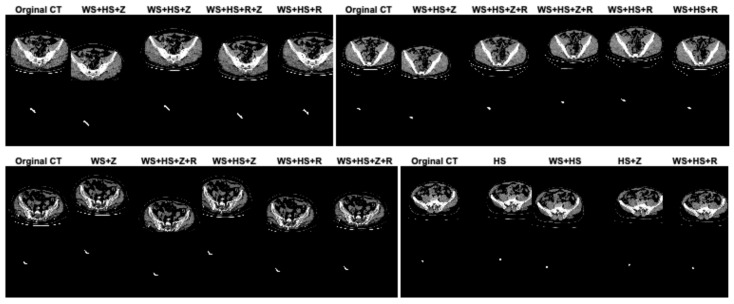
The effect of the data augmentation procedures on some CT scans and GT masks in the dataset.

In this study, data augmentation was performed to address several key challenges in training deep learning models for appendix segmentation in CT scans. Data augmentation was applied to artificially increase the size and variability of the prepared dataset. This helps to avoid overfitting [38], where the model memorizes the training data rather than learning generalizable features. In addition, the CT scans in the dataset can vary significantly between patients in terms of anatomical differences, scan quality, patient position and image noise. The data augmentation enables the proposed U-Net deep learning architecture to learn more robust and discriminative features. This improves the model’s ability to generalize to unseen data by simulating variations in real CT scans. In this study, in the original dataset prepared for appendix segmentation, the number of slices in the training set was increased from 1199 to 2399, the number of CT slices in the test set was increased from 221 to 441, and the total number of slices was increased from 1420 to 2840 using data augmentation strategies. The data augmentation procedures used to increase the size of the dataset were width_shift_range = 0.2 (WS), height_shift_range = 0.2 (HS), rotation_range = 2 (R) and zoom_range = 0.05 (Z). The data augmentation procedures for the dataset were randomly generated on both training and test sets separately or together. Figure 4 shows the effect of the data augmentation procedures on some CT scans and GT masks in the dataset.

The proposed U-Net model for appendix segmentation was subjected to training processes in the working environment using the original dataset obtained after data augmentation. In the original version of the dataset, a total of 1420 slices were collected from CT scans. Using the data augmentation procedures, the total number of CT slices in the dataset was doubled, resulting in a total of 2840 CT slices. For appendix segmentation, 2399 (~84%) of the 2840 images were allocated to the training phase and the remaining 441 (~16%) to the test phase.

In this study, the results of the proposed U-Net architecture for appendix detection from CT scans are also compared with the results of the state-of-the-art DenseNet [39] and Residual U-Net (Res U-Net) [40] architectures. The most prominent feature of DenseNet is that each layer of the network is directly connected to the outputs of all the previous layers and is widely used in segmentation problems. Res U-Net is a variation of the classical U-Net model where residual links are used as a basis to increase the depth of the model.

**Figure 5:**
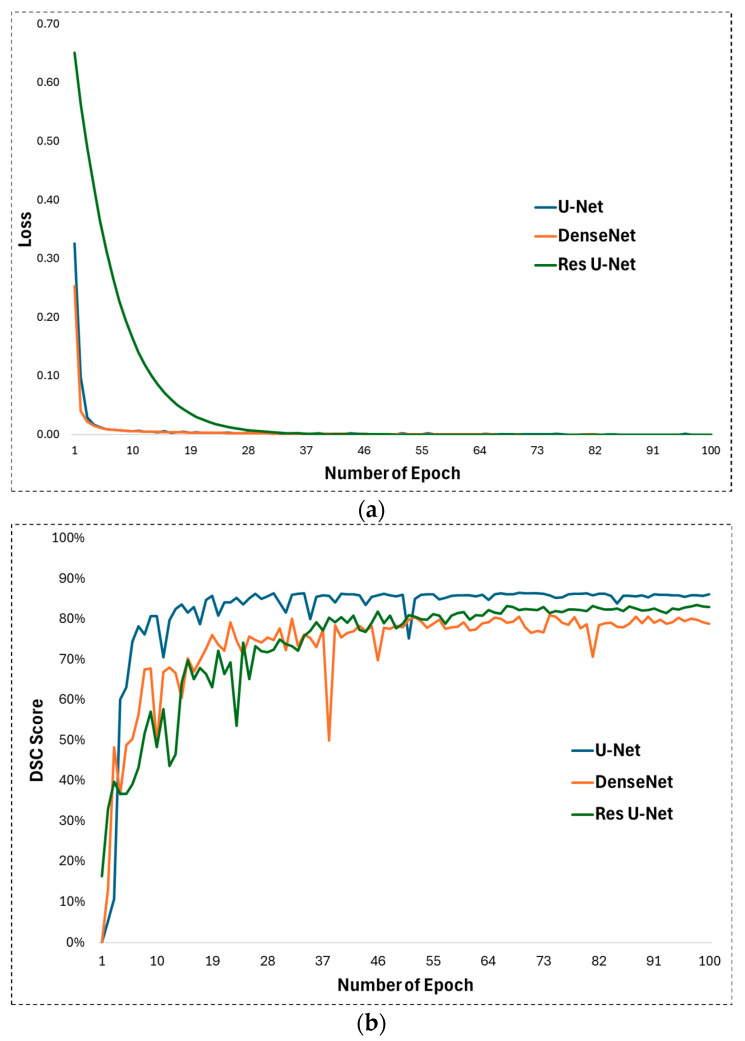
(**a**) Training loss and (**b**) DSC development during test phase for U-Net, DenseNet, and Res U-Net methods.

In experimental studies of this study, some hyperparameters in the U-Net architecture proposed for appendix segmentation are optimized and their optimal values for high segmentation performance are determined. First, ReLU is used as the activation function in the U-Net architecture. ReLU allows the U-Net to learn and model complex, non-linear mappings between input images and output segmentations. On the other hand, the Adam function was chosen as the optimization function. The Adam optimizer is well fitted to train the U-Net as it handles the complex nature of the deep layers of the architecture with adaptive learning rates and impulse-like behaviour. The learning rate was set to 0.001 for training the U-Net network. The learning rate plays a significant role in how quickly or slowly the U-Net model converges to an optimal solution as it learns from the data, especially for image segmentation tasks such as appendix detection in CT scans. Training was performed with these parameters for 100 epochs. The development of the loss during the training process is shown in Figure 5a. Looking at this figure, it is clear that the training process achieved a continuous improvement. During the training process, the weights after 100 epochs were obtained and subjected to the test phase. The DSC evolution for the 100 epochs can be seen in Figure 5b, which illustrates the performance during testing. It can be seen that the DSC value reaches an effective level after the first 10 epochs.

**Figure 6:**
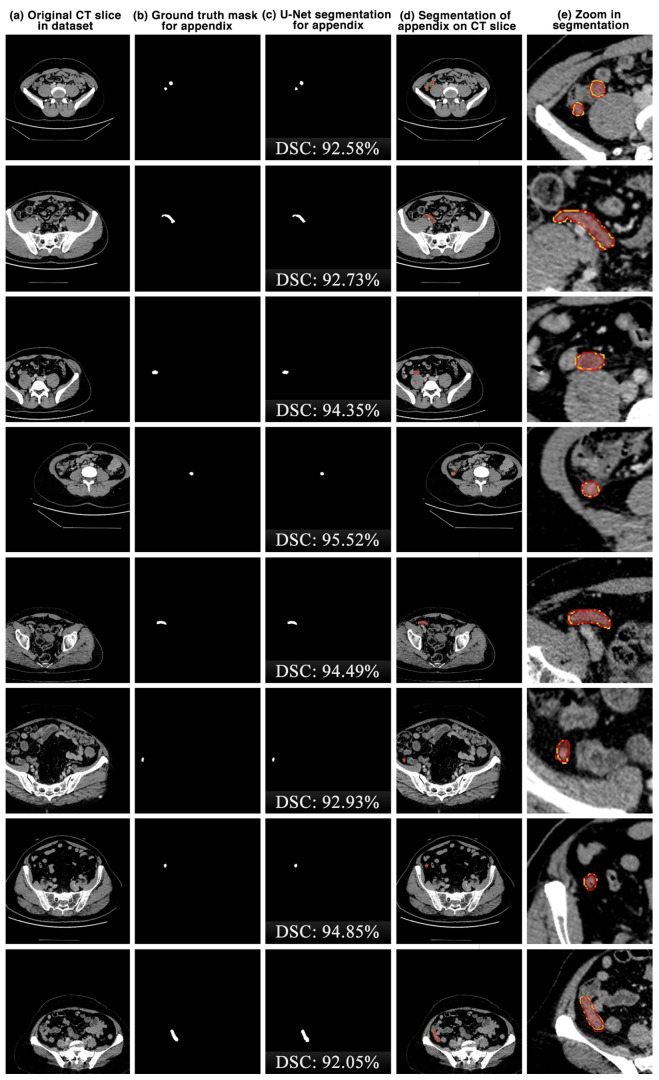
The appendix regions successfully detected and segmented on CT slices using the proposed U-Net deep learning architecture during the experimental studies. Red: ground truth mask for appendix, yellow: U-Net segmentation for appendix.

**Figure 7:**
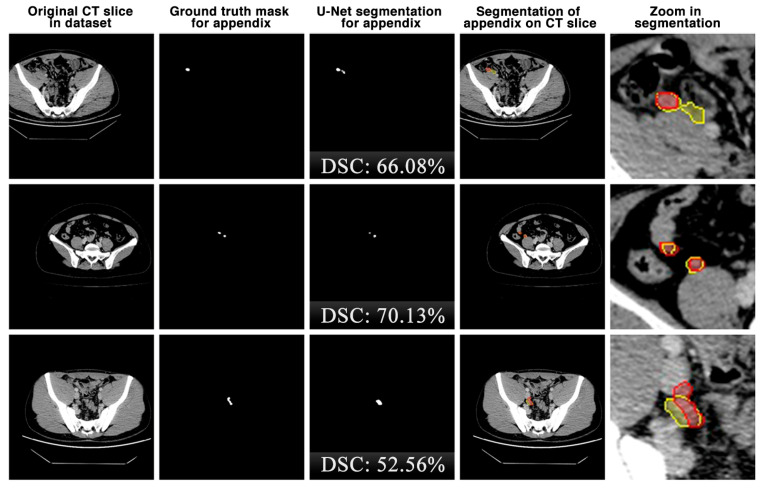
Some examples of unsuccessful appendix detection and segmentation by the proposed U-Net model. Red: ground truth mask for appendix, yellow: U-Net segmentation for appendix.

The appendix regions successfully detected and segmented on CT slices using the proposed U-Net deep learning architecture during the experimental studies are shown in Figure 6. While the mask of an original CT slice in Figure 6a containing the appendix region delineated by the expert is shown in Figure 6b, the segmentation of the appendix by the proposed U-Net deep learning architecture is shown in Figure 6c along with the DSC score. On the other hand, the overlap of the expert mask and the U-Net segmentation on the CT slice is also shown in Figure 6d and its zoomed version is shown in Figure 6e. As can be seen, the proposed U-Net-based deep learning architecture is very successful in segmenting the appendix CT scans and is very close to the expert GTs. Some examples of the segments that the proposed model has difficulty in detecting are shown in Figure 7. When analysing Figure 7, it is clear that the model cannot successfully detect some slices. The lack of success in these slices can be attributed to the fact that the appendix region is close to or adjacent to other areas, or the boundaries of the region are similar to those of the neighbouring areas. Such cases stood out as instances where the boundaries of the appendix were blurred and the model was unstable.

## 4. Discussion

The performance metrics results obtained using the proposed U-Net deep learning architecture and other state-of-the-art models for appendix segmentation are shown in Table 1. The proposed U-Net model is compared with other previous deep learning-based methods. Our model is particularly successful in accurately segmenting small and ambiguous patch boundaries. Comparisons with other methods show that U-Net provides better performance due to its encoder–decoder structure and the ability of skip connections to preserve spatial information. For DSC, which is the most important key metric for appendix segmentation performance, the proposed U-Net architecture achieves a score of 86.58%, while the Res U-Net and DenseNet architectures achieves 83.53% and 80.64% for the same metric, respectively. For the other key metrics with 22.99% for VOE, 1.08 mm for ASSD, 3.87 mm for HD95, and 87.08% for REC, it can be concluded that the U-Net architecture is more successful than Res U-Net and DenseNet. In addition, it is seen that DenseNet architecture is more successful with 88.56% only for PRE key metric.

**Figure 8:**
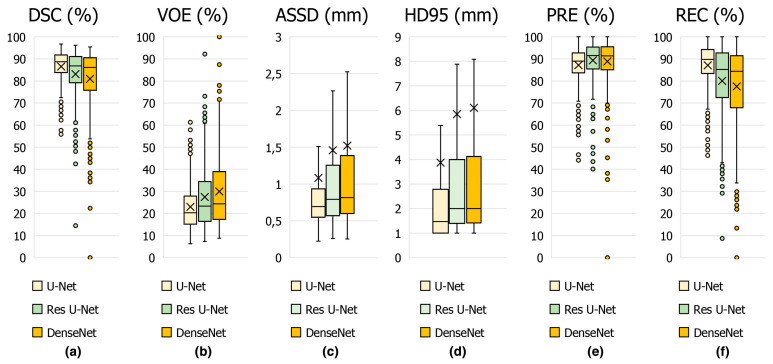
Boxplot showing the performance metrics for appendix segmentation obtained using the proposed U-Net deep learning architecture and other models in the study.

In Figure 8, the average scores achieved on the test set of CT slices for the proposed U-Net and other state-of-the-art methods such as DenseNet and Res U-Net are represented by box plots in terms of DSC in Figure 8a, VOE in Figure 8b, ASSD in Figure 8c, HD95 in Figure 8d, PRE in Figure 8e and REC in Figure 8f key metrics. From these results, it can be concluded that the proposed U-Net-based deep learning model is highly successful in detecting the appendix. Furthermore, our dataset, which was annotated by expert physicians and included CT scans from both healthy individuals and patients, provided high-quality and diverse training data, which enhanced the generalization of the U-Net model. We used hyper-parameter optimization with the ReLu activation function and the ADAM optimizer, which are known for efficient training and convergence [41,42]. These choices, combined with comprehensive evaluation metrics such as DSC, VOE, ASSD and HD95, ensured detailed evaluation and high performance. Such differences in architecture, data quality, augmentation, and evaluation strategies are likely to contribute to U-Net model’s competitive DSC of 86.58% in appendix detection, highlighting the importance of these factors in achieving superior diagnostic performance.

Due to the absence of previous studies specifically focusing on appendix segmentation, it was not possible to compare our study’s results directly with existing studies on this topic. However, comparisons can be drawn from similar tasks in the appendix such as segmentation, diagnosis, and classification, as shown in Table 2. In previous studies, classification research has generally been carried out using different CNN architectures in the diagnosis of appendicitis [15,43,44,45]. However, it is very important to determine the location of the appendix from the scans in cases. Therefore, in this study, the segmentation of the appendix is first achieved with a high score, and an important step in the diagnosis is completed. These comparative analyses help highlight the potential and robustness of our proposed model for appendix segmentation, suggesting its promising application in this area.

Automated appendix detection using U-Net deep learning architecture has some significant advantages and some disadvantages. On the positive side, these systems are much faster than manual methods, saving valuable time and enabling faster diagnoses. They offer high accuracy and consistency because they are trained on large datasets, minimizing human error. In addition, deep learning models can be customized for different patient populations and imaging modalities, resulting in more personalized and accurate analyses. These systems can process large amounts of data, improving their performance over time, and are able to detect small abnormalities in the early stages of appendicitis, allowing for earlier treatment. However, there are challenges to consider. The performance of deep learning models is highly dependent on the quality and diversity of the training data. Poor quality or insufficient datasets can lead to inaccurate results. Developing and implementing these systems can be expensive, both initially and in terms of ongoing maintenance and updates. The complexity of building and training these models requires specialized expertise, which may require the hiring of skilled personnel. There is also a risk of misleading results, particularly in rare cases or situations not represented in the training data, which can lead to false positives or false negatives. Finally, ensuring the privacy and security of medical data is critical, and robust protocols must be in place to protect this sensitive information. In conclusion, while the fully automated segmentation of the appendix region using deep learning models can significantly improve the speed and accuracy of medical diagnoses, its success depends largely on the quality of the data and the continuous improvement of the system.

**Figure 9:**
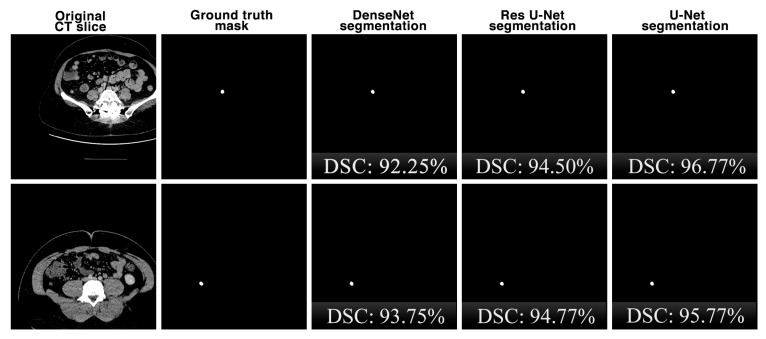
The comparison of the appendix segmentation performances of the proposed U-Net deep learning model and other state-of-the-art DenseNet and Res U-Net architectures on the same CT slices.

Figure 9 shows the comparison of the segmentation performances of the proposed U-Net deep learning model and other state-of-the-art DenseNet and Res U-Net architectures on the same CT slices in the dataset in terms of the DSC key metric. Although the performances of the architectures are close to each other for the segmentation results, it is seen that the U-Net architecture is slightly more successful.

## 5. Conclusions

Our study showed the significant potential of using a U-Net-based deep learning model for the accurate segmentation and detection of the appendix in CT images. With an achieved DSC score of 86.58%, the model shows promise in aiding clinical diagnosis and potentially improving patient outcomes. Detailed annotation by expert physicians and extensive data augmentation techniques contributed to the robustness and reliability of the model. The results of this study, demonstrating high performance in the detection of appendicitis using a U-Net-based deep learning model, have significant potential clinical implications. Improved diagnostic accuracy can reduce misdiagnosis and prevent complications such as perforation and sepsis, while the reduction in diagnostic time enables faster treatment decisions. The model’s ability to provide consistent and standardized diagnoses reduces clinician variability and human error. In addition, the model’s rapid and accurate diagnosis can optimize healthcare resources by minimizing unnecessary imaging and surgery. As a decision support tool, the model can assist radiologists by highlighting areas of interest and providing second opinions, which is particularly beneficial for less experienced practitioners. Early and accurate detection allows for timely intervention, improving patient outcomes. Integrating the U-Net model into clinical workflows can streamline the diagnostic process, and further research could extend its applications by refining the model and incorporating multimodal data to improve its accuracy. Overall, the robust performance of the model highlights the transformative potential of deep learning in medical diagnostics, particularly in the timely and accurate diagnosis of appendicitis.

To further improve our appendix detection model, we can focus on expanding and diversifying the dataset by including more CT scans from a varied patient population and multiple medical centres to improve generalization. Advanced data augmentation techniques, such as 3D augmentation and the use of generative adversarial networks for synthetic data generation, can introduce additional variability. Improving the model architecture by integrating hybrid models, using transfer learning, and applying ensemble methods can also improve performance. Automated hyperparameter tuning and advanced regularization techniques can optimize model parameters and prevent overfitting. The integration of clinical and imaging data through multimodal fusion techniques can provide a more comprehensive input for improved accuracy. Finally, continuous evaluation in real-world clinical settings and regular model updates with new data can ensure that the model remains accurate and relevant, ultimately leading to better diagnostic outcomes and improved patient care. To further improve model performance, future work should focus on expanding the dataset with more diverse and comprehensive samples, employing advanced augmentation and hybrid modelling techniques, and integrating multimodal data. In addition, the incorporation of explicable AI methods may improve the interpretability and trustworthiness of the model. Continuous evaluation in clinical settings and regular updates with new data can ensure that the model remains accurate and relevant. These advances can solidify the role of AI in medical diagnostics, ultimately leading to more precise and timely treatments for patients.

## Figures and Tables

**Table 1:** The comparison of the results of the proposed U-Net architecture with the results of the state-of-the-art DenseNet and Res U-Net architectures for the detection of the appendix from CT scans.

Method	DSC [%]	VOE [%]	ASSD [mm]	HD95 [mm]	PRE [%]	REC [%]
DenseNet	80.64	30.12	1.49	5.84	88.56	77.49
Res U-Net	83.53	26.99	1.73	6.93	88.06	81.51
Proposed U-Net	86.58	22.99	1.08	3.87	87.08	87.08

**Table 2:** Comparison of previous studies for automatic segmentation of appendix and classification of appendicitis.

Study	Year	Number of Images/Subjects	Research Topic	Methodology	Key Metric Evaluation (%)
Al et al. [43]	2019	319 CT examinations	Classification of acute appendicitis	Reinforcement Learning and CNN	AUC = 96.1
Rajpurgar et al. [15]	2020	646 CT examinations	Classification of appendicitis	3D CNN	AUC = 82.6
Park et al. [44]	2020	667 CT images	Classification of appendicitis	3D CNN	AUC = 96.0
Park et al. [45]	2023	4078 CT images	Classification of acute appendicitis, diverticulitis, and normal appendix	CNN (EfficientNet)	AUC = 95.1 (acute appendicitis) AUC = 97.2 (acute diverticulitis) AUC = 97.9 (normal appendix)
Ours	2024	940 CT images	Segmentation of appendix	U-Net-based deep learning architecture with hyperparameter optimization	DSC = 86.58

## Data Availability

The raw data supporting the conclusions of this article will be made available by the authors on request.
